# Supplementary material for: Evolution of schooling drives changes in neuroanatomy and motion characteristics across predation contexts in guppies
Source: Nat Commun. 2023 Sep 27;14:6027. doi: 10.1038/s41467-023-41635-6 (PMC10533906; doi:10.1038/s41467-023-41635-6)
Supplement: Supplementary file 1 — Supplementary Information [file 41467_2023_41635_MOESM1_ESM.pdf]

SUPPLEMENTARY FILE

Evolution of schooling drives changes in neuroanatomy and motion characteristics across predation contexts in guppies

Alberto Corral-Lopez*, Alexander Kotrschal, Alexander Szorkovszky, Maddi Garate-Olaizola, James Herbert-Read, Wouter van der Bijl, Maksym Romenskyy, Hong-Li Zeng, Severine Denise Buechel, Ada Fontrodona-Eslava, Kristiaan Pelckmans, Judith E. Mank, Niclas Kolm

*corresponding author: alberto.corral@ebc.su.se

SUPPLEMENTARY TABLES

Supplementary Table 1. Results from Linear Mixed Models evaluating potential differences in groups of polarization-selected and control female guppies in their shoaling patterns when exposed to an open field test (OFT), a novel object (cup) and a predator model (see Supplementary code on shoaling patterns analyses). In bold p-values lower than 0.05.

Polarization

Predictors	*Estimates*	*CI*	*t*	*p*	*df*
(Intercept)	0.80	0.68 – 0.91	14.54	**< 0.001**	17.59
Selection [P]	0.08	0.02 – 0.13	3.90	**0.017**	3.98
treatment [Cup]	-0.12	-0.15 – -0.10	-9.16	**< 0.001**	338.42
treatment [Predator]	-0.17	-0.20 – -0.14	-12.61	**< 0.001**	340.73
Body size	-0.00	-0.00 – -0.00	-2.35	**0.021**	88.01
Selection [P] * treatment [Cup]	-0.01	-0.05 – 0.03	-0.46	0.644	340.46
Selection [S] * treatment [Predator]	-0.00	-0.04 – 0.03	-0.18	0.860	341.08

Median speed

Predictors	*Estimates*	*CI*	*t*	*p*	*df*
(Intercept)	63.25	46.15 – 80.34	7.50	**< 0.001**	35.87
Selection [P]	13.63	8.09 – 19.16	5.96	**< 0.001**	6.29

treatment [Cup]	-20.14	-23.91 – -16.37	-10.51	**< 0.001**	337.87
treatment [Predator]	-24.34	-28.14 – -20.54	-12.60	**< 0.001**	339.82
Body size	-0.06	-0.12 – 0.01	-1.77	**0.037**	85.64
Selection [P] * treatment [Cup]	-4.55	-9.85 – 0.76	-1.69	0.093	339.52
Selection [S] * treatment [Predator]	-3.95	-9.27 – 1.36	-1.46	0.144	340.05

Nearest neighbor distance

Predictors	*Estimates*	*CI*	*t*	*p*	*df*
(Intercept)	39.17	33.89 – 44.45	14.96	**< 0.001**	43.22
Selection [P]	-3.51	-5.34 – -1.53	-4.55	**0.007**	4.83
treatment [Cup]	-10.77	-11.94 – -9.60	-18.13	**< 0.001**	337.80
treatment [Predator]	-10.88	-12.06 – -9.70	-18.17	**< 0.001**	339.69
Body size	-0.01	-0.03 – 0.01	-0.63	0.460	105.88
Selection [P] * treatment [Cup]	2.88	1.24 – 4.53	3.45	**< 0.001**	339.38
Selection [S] * treatment [Predator]	2.82	1.17 – 4.47	3.37	**< 0.001**	339.88

Supplementary Table 2. Independent contrasts of Linear Mixed Models for comparisons between polarization-selected and control female guppies in their shoaling patterns when exposed to an open field test (OFT), a novel object (cup) and a predator model. In bold p-values lower than 0.05 with Tukey p-value adjustment for multiple comparisons (see Supplementary code on shoaling patterns analyses).

Polarization

contrast	*treatment*	*estimate*	*SE*	*df*	*lower.CL*	*upper.CL*	*t.ratio*	*p.value*
C - P	OFT	-0.077	0.020	3.980	-0.133	-0.022	-3.898	**0.018**
C - P	Cup	-0.069	0.020	4.078	-0.124	-0.014	-3.435	**0.026**
C - P	Predator	-0.074	0.020	4.106	-0.129	-0.019	-3.698	**0.020**

Median speed

contrast	*treatment*	*estimate*	*SE*	*df*	*lower.CL*	*upper.CL*	*t.ratio*	*p.value*
C - P	OFT	-13.627	2.288	6.292	-19.164	-8.091	-5.956	**0.001**
C - P	Cup	-9.079	2.311	6.560	-14.619	-3.538	-3.928	**0.006**
C - P	Predator	-9.673	2.321	6.618	-15.226	-4.121	-4.168	**0.005**

Nearest neighbor distance

contrast	*treatment*	*estimate*	*SE*	*df*	*lower.CL*	*upper.CL*	*t.ratio*	*p.value*
C - P	OFT	3.434	0.754	5.309	1.530	5.339	4.555	**0.005**
C - P	Cup	0.552	0.761	5.510	-1.350	2.455	0.726	0.498
C - P	Predator	0.613	0.764	5.560	-1.292	2.517	0.802	0.455

Supplementary Table 3. Results from Generalized Linear Mixed Models evaluating potential differences in predator inspection behavior of polarization-selected and control female guppy groups in experiments exposing them to a predator model. In bold p-values lower than 0.05 (see Supplementary code on predator inspection analyses)

Number of inspections

Predictors	*Incidence Rate Ratios*	*CI*	*t*	*p*
(Intercept)	4.47	3.64 – 5.50	14.19	**< 0.001**
Selection [P]	0.87	0.75 – 1.01	-1.85	0.0645

Total time inspecting

Predictors	*Incidence Rate Ratios*	*CI*	*t*	*p*
(Intercept)	24.04	18.02 – 32.07	21.64	**< 0.001**
Selection [P]	0.79	0.66 – 0.95	-2.52	**0.011**

Mean inspection duration

Predictors	*Incidence Rate Ratios*	*CI*	*t*	*p*
(Intercept)	5.62	4.84 – 6.53	22.63	**< 0.001**
Selection [P]	0.82	0.69 – 0.96	-2.49	**0.013**

Supplementary Table 4a. Results from a Linear Mixed Model evaluating potential differences in group polarization of polarization-selected and control female guppy groups when the average position of the group was shorter than 200 mm to the stimulus presented in the arena in tests that exposed these fish to a predator model and a novel object (cup). In bold p-values lower than 0.05 csee Supplementary code on analyses of group polarization with a predator model)

**Polarization (closer than 200 mm from the predator model)**					
Predictors	*Estimates*	*CI*	*t*	*p*	*df*
(Intercept)	0.68	0.60 – 0.75	25.78	**< 0.001**	3.87
Selection [P]	0.07	0.02 – 0.12	2.96	**0.003**	273.80
treatment [Predator model]	-0.12	-0.16 – -0.08	-5.34	**< 0.001**	146.76
Replicate [Rep2]	0.00	-0.04 – 0.04	0.05	0.958	143.72
replicate [Rep3]	0.01	-0.03 – 0.05	0.32	0.749	142.38
Selection [P] * treatment [Predator model]	-0.03	-0.08 – 0.03	-0.83	0.405	143.99

Supplementary Table 4b. Independent contrasts of a Linear Mixed Model evaluating group polarization of polarization-selected and control female guppies when swimming at a distance closer than 200 mm to the stimulus presented in the arena in tests that exposed these fish to a predator model and a novel object (cup). In bold p-values lower than 0.05 with Tukey p-value adjustment for multiple comparisons (see Supplementary code on analyses of group polarization with a predator model).

contrast	*treatment*	*estimate*	*SE*	*df*	*lower.CL*	*upper.CL*	*t.ratio*	*p.value*
C - P	Cup	-0.070	0.024	273.79	-0.116	-0.023	-2.956	**0.003**
C - P	Predator	-0.044	0.022	272.27	-0.088	-0.000	-1.984	**0.048**

Supplementary Table 5a. Results from a Linear Mixed Model evaluating potential differences in group polarization of polarization-selected and control female guppies when the average position of the group was located closer to the tail area or closer to the head area of the predator model. In bold p-values lower than 0.05 (see Supplementary code on analyses of group polarization with a predator model)

**Polarization**					
Predictors	*Estimates*	*CI*	*Statistic*	*p*	*df*
(Intercept)	0.62	0.54 – 0.71	22.54	< **0.001**	3.15
Selection [P]	0.04	-0.03 – 0.10	1.53	0.191	4.58
location [tail]	-0.11	-0.14 – -0.07	-5.47	< **0.001**	156.96
line [Social] * location [tail]	0.04	-0.01 – 0.09	1.64	0.103	150.99

Supplementary Table 5b. Independent contrasts of a Linear Mixed Model evaluating group polarization of polarization-selected and control female guppy groups when the average position of the group was located closer to the tail area or closer to the head area of the predator model. In bold p-values lower than 0.05 with Tukey p-value adjustment for multiple comparisons (see Supplementary code on analyses of group polarization with a predator model).

contrast	*location*	*estimate*	*SE*	*df*	*lower.CL*	*upper.CL*	*t.ratio*	*p.value*
C - P	Head	-0.037	0.024	4.58	-0.102	0.027	-1.534	0.191
C - P	Tail	-0.080	0.023	3.63	-0.146	-0.013	-3.483	**0.030**

Supplementary Table 6. Results from Linear Mixed Models (LMMs) evaluating differences in relative brain and relative brain region size between polarization-selected and control female guppies. In bold p-values lower than 0.05. Results are based on separate models for each brain region, without multiple testing p-value correction (see Supplementary code on neuroanatomy analyses).

Region	Coefficent	Estimate	SE	df	t	P-value
Whole brain	Intercept	10.706	0.929	22.992	11.520	**< 0.001**
	Sel. Line (Polarization)	-0.015	0.037	23.296	-0.415	0.682
	Log (SL)	1.581	0.292	22.899	5.401	**< 0.001**
Olfactory bulbs	Intercept	-5.639	4.048	24.311	-1.393	0.176
	Sel. Line (Polarization)	-0.054	0.071	23.443	-0.759	0.455
	Log (rest of brain)	0.973	0.257	24.319	3.782	**< 0.001**
Ventral telencephalon	Intercept	-7.112	4.022	23.970	-1.768	0.0897
	Sel. Line (Polarization)	-0.054	0.070	23.366	-0.762	0.453
	Log (rest of brain)	1.107	0.255	23.968	4.329	**< 0.001**
Dorsal telencephalon	Intercept	-3.002	1.502	25.000	-1.998	0.0567
	Sel. Line (Polarization)	0.029	0.027	25.000	1.096	0.2837
	Log (rest of brain)	0.935	0.095	25.000	9.780	**< 0.001**
Thalamus	Intercept	-1.019	1.805	25.000	-0.565	0.577
	Sel. Line (Polarization)	0.073	0.033	25.000	2.187	**0.038**
	Log (rest of brain)	0.950	0.115	25.000	8.197	**< 0.001**
Hypothalamus	Intercept	-3.858	2.745	25.000	-1.405	0.172
	Sel. Line (Polarization)	0.048	0.049	25.000	0.974	0.339
	Log (rest of brain)	1.098	0.175	25.000	6.259	**< 0.001**
Nucleus glomerulus	Intercept	-2.352	9.716	25.000	-0.242	0.811
	Sel. Line (Polarization)	-0.067	0.176	25.000	-0.381	0.706
	Log (rest of brain)	0.807	0.617	25.000	1.308	0.203
Torus semicircularis	Intercept	-0.665	2.368	24.977	-0.281	0.781
	Sel. Line (Polarization)	-0.0325	0.042	24.106	-0.758	0.456
	Log (rest of brain)	0.829	0.150	24.982	5.496	**< 0.001**
Optic tectum cup	Intercept	1.073	0.966	23.494	1.111	0.2780
	Sel. Line (Polarization)	0.042	0.017	23.095	2.409	**0.024**
	Log (rest of brain)	0.800	0.061	23.474	12.933	**< 0.**001
Optic tectum core	Intercept	-2.883	1.658	25.000	-1.739	0.0944
	Sel. Line (Polarization)	0.006	0.030	25.000	0.211	0.8347
	Log (rest of brain)	1.040	0.106	25.000	9.805	**< 0.001**
Cerebellum	Intercept	-0.403	1.458	24.350	-0.277	0.784
	Sel. Line (Polarization)	-0.007	0.026	23.221	-0.275	0.786
	Log (rest of brain)	0.908	0.093	24.358	9.703	**< 0.001**
Medulla oblongata	Intercept	-0.145	1.519	24.920	-0.096	0.9246
	Sel. Line (Polarization)	-0.074	0.028	23.916	-2.656	**0.013**
	Log (rest of brain)	0.969	0.099	24.935	9.761	**< 0.001**

Supplementary Table 7a. Results from a Bayesian multilevel model evaluating differences in relative brain region size between polarization-selected and control female guppies. Stars and values in bold indicate estimates that do not include zero in the confidence interval range based on the posterior samples drawn from the model (see Supplementary code on neuroanatomy analyses).

Covariate	***Estimate***	***Est.Error***	***l.95..CI***	***u.95..CI***	
Medulla_Intercept	0.19	0.32	-0.37	0.77	
Cerebellum_Intercept	0.05	0.50	-1.08	1.04	
Nucleus glomerulus_Intercept	0.09	0.48	-0.84	1.04	
Torus semicircularis_Intercept	0.14	0.44	-0.69	1.03	
Thalamus_Intercept	-0.25	0.34	-0.97	0.31	
Optic tectum cups_Intercept	-0.19	0.51	-1.26	0.89	
Hypothalamus_Intercept	-0.06	0.39	-0.84	0.73	
Olfactory bulbs_Intercept	0.13	0.54	-0.94	1.26	
Ventral telencephalon_Intercept	0.05	0.63	-1.23	1.43	
Dorsal telencephalon_Intercept	-0.09	0.32	-0.72	0.56	
Optic tectum core_Intercept	-0.02	0.28	-0.56	0.54	
Medulla oblongata_Selection	-0.42	0.18	**-0.79**	**-0.06**	*
Medulla oblongata_Rest of the brain	0.91	0.09	**0.74**	**1.09**	*
Cerebellum_Selection	-0.07	0.21	-0.48	0.35	
Cerebellum_Rest of the brain	0.96	0.11	**0.75**	**1.18**	*
Nucleus glomerolus_Selection	-0.21	0.40	-0.98	0.58	
Nucleus glomerulus_Rest of the brain	0.34	0.20	-0.06	0.75	
Torus semicircularis_Selection	-0.24	0.32	-0.87	0.38	
Torus semicircularis_Rest of the brain	0.72	0.16	**0.40**	**1.04**	*
Thalamus_Selection	0.49	0.23	**0.04**	**0.94**	*
Thalamus_Rest of the brain	0.92	0.11	**0.70**	**1.16**	*
Optic tectum cups_Selection	0.32	0.16	**0.00**	**0.63**	*
Optic tectum cups_Rest of the brain	0.88	0.08	**0.72**	**1.06**	*
Hypothalamus_Selection	0.12	0.26	-0.39	0.64	

Hypothalamus_Rest of the brain	0.87	0.13	**0.61**	**1.14**	*
Olfactory bulbs _Selection	-0.27	0.33	-0.93	0.38	
Olfactory bulbs_Rest of the brain	0.62	0.17	**0.29**	**0.95**	*
Ventral telencephalon_Selection	-0.17	0.31	-0.76	0.43	
Ventral telencephalon_Rest of the brain	0.58	0.16	**0.27**	**0.89**	*
Dorsal telencephalon_Selection	0.18	0.21	-0.25	0.60	
Dorsal telencephalon_Rest of the brain	0.91	0.11	**0.69**	**1.13**	*
Optic tectum core_Selection	0.05	0.20	-0.34	0.44	
Optic tectum core_Rest of the brain	0.93	0.10	**0.73**	**1.13**	*

Supplementary Table 7b. Residual correlations of thalamus, optic tectum cups and medulla oblongata relative volume to other brain regions estimated from a Bayesian multilevel model evaluating differences in relative brain region size between polarization-selected and control female guppies. Stars and values in bold indicate estimates that do not include zero in the confidence interval range based on the posterior samples drawn from the model (see Supplementary code on neuroanatomy analyses).

Medulla oblongata

Brain region	*Estimate*	*Est.Error*	*l.95..CI*	*u.95..CI*	
Cerebellum	-0.32	0.15	**-0.59**	**-0.02**	*
Nucleus glomerulus	-0.26	0.15	-0.53	0.05	
Torus semicircularis	0.07	0.16	-0.25	0.37	
Thalamus	-0.40	0.14	**-0.65**	**-0.12**	*
Optic tectum cups	-0.04	0.17	-0.36	0.29	
Hypothalamus	-0.50	0.12	**-0.72**	**-0.23**	*
Olfactory bulbs	-0.24	0.16	-0.52	0.09	
Ventral telencephalon	-0.15	0.16	-0.44	0.17	
Dorsal telencephalon	-0.12	0.17	-0.43	0.21	
Optic tectum core	-0.22	0.15	-0.51	0.09	

Optic tectum cups

Brain region	*Estimate*	*Est.Error*	*l.95..CI*	*u.95..CI*
Medulla oblongata	-0.04	0.16	-0.36	0.28
Cerebellum	-0.07	0.17	-0.41	0.26
Nucleus glomerulus	-0.07	0.17	-0.40	0.26
Torus semicircularis	0.05	0.17	-0.29	0.38
Thalamus	-0.18	0.16	-0.49	0.15

Hypothalamus	-0.15	0.16	-0.46	0.17
Olfactory bulbs	-0.11	0.17	-0.45	0.23
Ventral telencephalon	0.02	0.17	-0.32	0.36
Dorsal telencephalon	-0.01	0.17	-0.36	0.33
Optic tectum core	-0.06	0.17	-0.39	0.27

Thalamus

Brain region	***Estimate***	***Est.Error***	***l.95..CI***	***u.95..CI***	
Medulla oblongata	-0.40	0.14	**-0.65**	**-0.12**	*
Cerebellum	-0.27	0.16	-0.55	0.05	
Nucleus glomerulus	0.05	0.16	-0.26	0.36	
Torus semicircularis	-0.27	0.16	-0.56	0.06	
Optic tectum cups	-0.18	0.17	-0.49	0.15	
Hypothalamus	-0.15	0.16	-0.44	0.18	
Olfactory bulbs	-0.09	0.16	-0.41	0.24	
Ventral telencephalon	0.27	0.16	-0.05	0.56	
Dorsal telencephalon	-0.05	0.17	-0.37	0.28	
Optic tectum core	0.10	0.16	-0.22	0.41	

Supplementary Table 8. Results from Linear Mixed Models evaluating potential differences in eye size between individuals from polarization-selected and control lines. In bold p-values lower than 0.05 (see Supplementary code for eye size analyses).

Eye diameter

Predictors	*Estimates*	*CI*	*Statistic*	*p*	*df*
(Intercept)	3.12	2.95 – 3.29	78.98	**< 0.001**	2.00
Selection [P]	-0.03	-0.24 – 0.19	-0.52	0.658	2.00

Eye diameter / body length

Predictors	*Estimates*	*CI*	*Statistic*	*p*	*df*
(Intercept)	0.12	0.11 – 0.12	69.97	**< 0.001**	2.00
Selection [P]	-0.00	-0.01 – 0.01	-0.13	0.906	2.00

Supplementary Table 9a. Results from a Linear Mixed Model evaluating potential differences in optomotor response between individuals from polarization-selected and control lines. In bold p-values lower than 0.05 (see Supplementary code for optomotor response analyses).

**Optomotor response**					
Predictors	*Estimates*	*CI*	*Statistic*	*p*	*df*
(Intercept)	0.10	0.03 – 0.17	3.16	**0.008**	12.06
Selection [P]	0.00	-0.08 – 0.09	0.11	0.913	12.88
Band width stimulus [0.28]	0.07	0.00 – 0.14	2.09	**0.036**	570.82
Band width stimulus [0.31]	0.15	0.08 – 0.21	4.20	**< 0.001**	569.22
Band width stimulus [0.35]	0.12	0.05 – 0.19	3.51	**< 0.001**	569.28
Band width stimulus [0.39]	0.18	0.12 – 0.25	5.26	**< 0.001**	574.04
Band width stimulus [0.44]	0.17	0.10 – 0.23	4.75	**< 0.001**	572.07
Optomotor response in static	0.56	0.49 – 0.63	14.92	**< 0.001**	531.69
Selection [P]* Band width stimulus [0.28]	0.01	-0.08 – 0.11	0.26	0.793	569.30
Selection [P]* Band width stimulus [0.31]	0.01	-0.09 – 0.10	0.11	0.915	569.21
Selection [P]* Band width stimulus [0.35]	0.03	-0.06 – 0.13	0.63	0.526	569.18
Selection [P]* Band width stimulus [0.39]	-0.03	-0.13 – 0.06	-0.64	0.520	569.16
Selection [P]* Band width stimulus [0.44]	0.01	-0.09 – 0.10	0.17	0.863	569.22

Supplementary Table 9b. Independent contrasts of overall response to stimulus bandwidth in a Linear Mixed Model evaluating optomotor response in polarization-selected and control female guppies. In bold p-values lower than 0.05 with Tukey p-value adjustment for multiple comparisons (see Supplementary code on optomotor response analyses).

contrast	*estimate*	*SE*	*df*	*lower.CL*	*upper.CL*	*t.ratio*	*p.value*

bw25 - bw28	-0.079	0.024	571.167	-0.149	-0.009	-3.236	**0.016**
bw25 - bw31	-0.148	0.024	569.618	-0.218	-0.079	-6.094	**< 0.001**
bw25 - bw35	-0.137	0.024	569.230	-0.207	-0.068	-5.637	**< 0.001**
bw25 - bw39	-0.168	0.025	577.744	-0.238	-0.097	-6.818	**< 0.001**
bw25 - bw44	-0.169	0.024	573.616	-0.239	-0.099	-6.918	**< 0.001**
bw28 - bw31	-0.069	0.024	569.653	-0.139	0.000	-2.852	0.051
bw28 - bw35	-0.058	0.024	570.338	-0.128	0.011	-2.390	0.161
bw28 - bw39	-0.089	0.024	571.492	-0.159	-0.019	-3.639	**0.004**
bw28 - bw44	-0.090	0.024	569.612	-0.160	-0.021	-3.712	**0.003**
bw31 - bw35	0.011	0.024	569.262	-0.058	0.081	0.460	0.997
bw31 - bw39	-0.019	0.025	574.211	-0.089	0.051	-0.791	0.969
bw31 - bw44	-0.021	0.024	571.155	-0.091	0.049	-0.858	0.956
bw35 - bw39	-0.031	0.025	575.985	-0.101	0.040	-1.245	0.814
bw35 - bw44	-0.032	0.024	572.350	-0.102	0.038	-1.315	0.777
bw39 - bw44	-0.002	0.024	569.833	-0.071	0.068	-0.064	1.000

Supplementary Table 9c. Independent contrasts for selection line response to multiple stimuli presented and evaluated with a Linear Mixed Model (see Supplementary code on optomotor response analyses).

contrast	*stimulus*	*estimate*	*SE*	*df*	*lower.CL*	*upper.CL*	*t.ratio*	*p.value*
C - P	bw25	-0.004	0.038	601.455	-0.078	0.070	-0.112	0.911
C - P	bw28	-0.017	0.038	601.441	-0.091	0.057	-0.456	0.649
C - P	bw31	-0.009	0.038	601.422	-0.083	0.064	-0.247	0.805
C - P	bw35	-0.035	0.038	601.457	-0.109	0.039	-0.938	0.349
C - P	bw39	0.027	0.038	601.458	-0.047	0.101	0.722	0.471
C - P	bw44	-0.013	0.038	601.452	-0.086	0.061	-0.340	0.734

Supplementary Table 10a. Results from Linear Mixed ModelS evaluating potential differences in visual temporal resolution between individuals from polarization-selected and control lines. In bold p-values lower than 0.05 (see Supplementary code for temporal resolution analyses).

Speed deviation from stimulus rotation

Predictors	*Estimates*	*CI*	*Statistic*	*p*	*df*
(Intercept)	8.38	3.23 – 13.53	5.31	**0.014**	2.87
Selection [P]	-0.46	-8.74 – 7.83	-0.19	0.863	2.64
Speed [24]	-5.93	-7.71 – -4.15	-6.54	**< 0.001**	4398.86
Speed [36]	-23.39	-25.17 – -21.61	-25.78	**< 0.001**	4398.86
Speed [45]	-49.74	-51.52 – -47.96	-54.79	**< 0.001**	4398.84
Rotation[Counterclockwise]	-7.11	-8.36 – -5.85	-11.08	**< 0.001**	4398.89
Selection [P] * Speed [24]	0.31	-2.17 – 2.79	0.25	0.805	4398.91
Selection [P] * Speed [36]	1.26	-1.22 – 3.74	0.99	0.320	4398.91
Selection [P] * Speed [45]	-0.48	-2.96 – 1.99	-0.38	0.701	4398.84
Selection [P] * Rotation[Counterclockwise]	1.29	-0.47 – 3.04	1.44	0.150	4398.98

Proportion of time following the stimulus

Predictors	*Estimates*	*CI*	*Statistic*	*p*	*df*
(Intercept)	0.87	0.80 – 0.94	39.43	**< 0.001**	3.12
Selection [P]	0.00	-0.14 – 0.15	0.10	0.928	2.52
Speed [24]	0.03	0.00 – 0.06	2.03	**< 0.001**	4398.95
Speed [36]	0.03	-0.00 – 0.05	1.83	0.067	4398.95
Speed [45]	-0.04	-0.06 – -0.01	-2.55	**0.018**	4398.92
Rotation[Counterclockwise]	-0.07	-0.09 – -0.05	-7.33	**< 0.001**	4398.98
Selection [P] * Speed [24]	0.01	-0.02 – 0.04	0.74	0.457	4399.06
Selection [P] * Speed [36]	-0.00	-0.04 – 0.03	-0.20	0.843	4398.99
Selection [P] * Speed [45]	-0.00	-0.04 – 0.04	-0.15	0.884	4398.99

Selection [P] * Rotation[Counterclockwise]	-0.01	-0.05 – 0.03	-0.62	0.536	4398.92

Supplementary Table 10b. Independent contrasts for speed deviation to stimulus rotation observed in a Linear Mixed Model evaluating visual temporal resolution in polarization-selected and control female guppies at multiple rotation speeds in clockwise and counterclockwise directions. In bold p-values lower than 0.05 (see Supplementary code for temporal resolution analyses).

Selection	*Speed*	*Rotation*	*emmean*	*SE*	*df*	*lower.CL*	*upper.CL*	*t.ratio*	*p.value*
C	14.4	Clockwise	8.379	1.577	2.874	3.233	13.526	5.313	**0.015**
P	14.4	Clockwise	7.922	1.938	2.485	0.966	14.879	4.089	**0.038**
C	24	Clockwise	2.447	1.577	2.872	-2.701	7.594	1.552	0.222
P	24	Clockwise	2.303	1.938	2.488	-4.650	9.256	1.188	0.336
C	36	Clockwise	-15.012	1.577	2.872	-20.160	-9.865	-9.520	**0.003**
P	36	Clockwise	-14.212	1.938	2.488	-21.164	-7.259	-7.332	**0.010**
C	45	Clockwise	-41.357	1.577	2.874	-46.503	-36.210	-26.223	**< 0.001**
P	45	Clockwise	-42.299	1.938	2.485	-49.255	-35.342	-21.830	**0.001**
C	14.4	Counterclockwise	1.272	1.577	2.873	-3.875	6.420	0.807	0.481
P	14.4	Counterclockwise	2.103	1.938	2.487	-4.850	9.057	1.085	0.372
C	24	Counterclockwise	-4.660	1.577	2.869	-9.809	0.489	-2.956	0.063
P	24	Counterclockwise	-3.517	1.939	2.491	-10.465	3.432	-1.814	0.186
C	36	Counterclockwise	-0.483	-22.119	1.577	2.869	-27.268	-14.030	**0.001**
P	36	Counterclockwise	-20.031	1.939	2.491	-26.979	-13.083	-10.331	**0.004**
C	45	Counterclockwise	-48.464	1.577	2.873	-53.611	-43.317	-30.731	**< 0.001**
P	45	Counterclockwise	-48.118	1.938	2.487	-55.071	-41.164	-24.827	**< 0.001**

Supplementary Table 11. Statistical results using a robust linear mixed model approach for comparisons in the proportion of time following the correct direction of the stimulus in visual temporal resolution assays between polarization-selected and control female guppies. In bold p-values lower than 0.05 (see Supplementary code for temporal resolution analyses).

**Proportion of time following the stimulus**					
Predictors	*Estimates*	*Std. Error*	*t-value*	*p*	*df*
(Intercept)	0.91	0.016	57.45	**< 0.001**	3.12
Selection [P]	-0.001	0.031	-0.04	0.971	2.52
Speed [24]	0.014	0.009	1.61	0.106	4398.95
Speed [36]	0.027	0.009	3.07	**0.001**	4398.95
Speed [45]	-0.010	0.009	-1.17	0**.240**	4398.92
Rotation[Counterclockwise]	-0.041	0.006	-6.51	**< 0.001**	4398.98
Selection [P] * Speed [24]	0.004	0.012	0.33	0.744	4399.06
Selection [P] * Speed [36]	0.004	0.012	0.33	0.741	4398.99
Selection [P] * Speed [45]	-0.009	0.012	-0.74	0.460	4398.99
Selection [P] * Rotation[Counterclockwise]	-0.001	0.009	-0.15	0.882	4398.92

Robustness weights for the residuals:
3546 weights are ~= 1. The remaining 974 ones are summarized as

Min.	1st Qu.	Median	Mean	3rd Qu.	Max.
0.199	0.328	0.518	0.557	0.773	0.999

Robustness weights for the random effects:
101 weights are ~= 1. The remaining 18 ones are summarized as

Min.	1st Qu.	Median	Mean	3rd Qu.	Max.
0.247	0.489	0.739	0.693	0.904	0.995

Rho functions used for fitting:
Residuals:
eff: smoothed Huber (k = 1.345, s = 10), sig: smoothed Huber, Proposal II (k = 1.345, s = 10)
Random Effects, variance component 1 (trial):
eff: smoothed Huber (k = 1.345, s = 10), vcp: smoothed Huber, Proposal II (k = 1.345, s = 10)
Random Effects, variance component 2 (rep):
eff: smoothed Huber (k = 1.345, s = 10), vcp: smoothed Huber (k = 1.345, s = 10)

SUPPLEMENTARY FIGURES

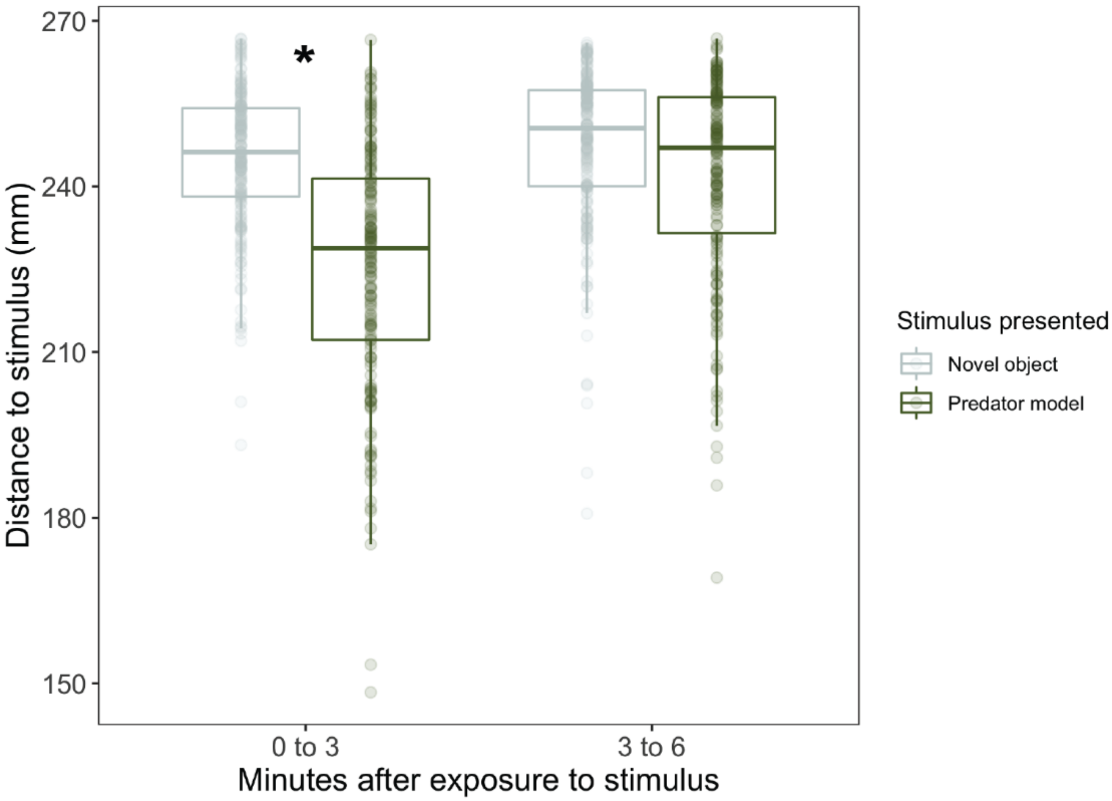

Supplementary Figure 1. Proximity to stimuli in female guppies artificially selected for higher polarization. Boxplots of median distance to the stimulus combining data for groups of polarization-selected (n = 89) and control (n = 85) female guppies in predator model (green) and novel object (gray) assays. Horizontal lines indicate medians, boxes indicate the interquartile range, and whiskers indicate all points within 1.5 times the interquartile range. Asterisk indicate a significant interaction in the distance of the average group position to the stimulus between time of the trial and type of stimulus presented obtained from a Linear Mixed Model (Estimate $_{\text{minutes x treatment}}$ = 13.87 ± 2.50, t = 5.55, df =499, p = 0.001) . Source data are provided as a Source Data file.

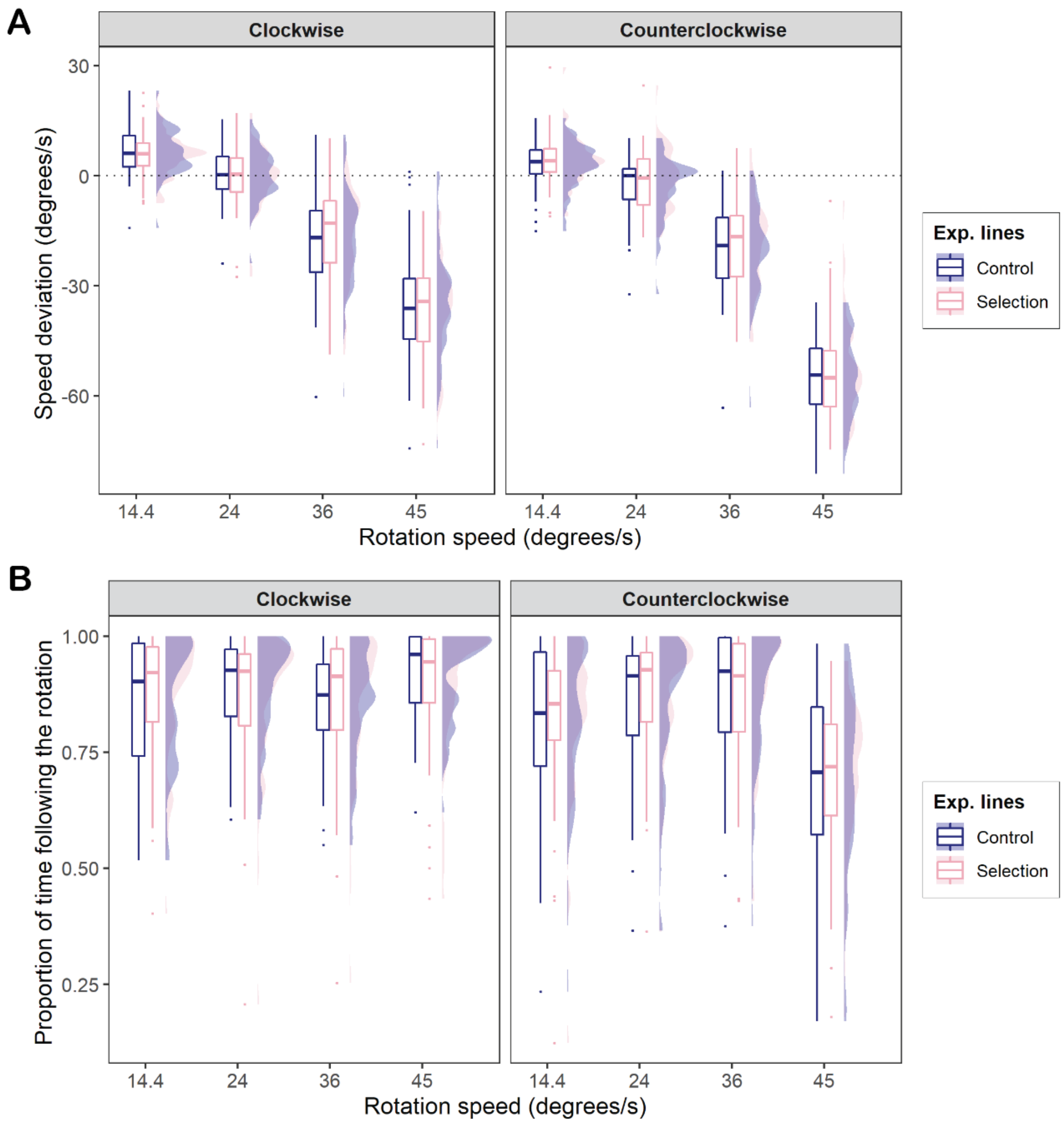

Supplementary Figure 2. Visual temporal resolution of female guppies artificially selected for higher polarization. Boxplots and density plots of the deviation of fish swimming speed **(A),** and the proportion of time that fish followed the direction of the stimulus **(B)** in relation to four different rotating stimuli presented at different speeds that rotated clockwise and anti-clockwise to polarization-selected (pink; n = 59) and control females (blue; n = 55). In the boxplots, horizontal lines indicate medians, boxes indicate the interquartile range, and whiskers indicate all points within 1.5 times the interquartile range. No significant differences were observed for any comparison between control and polarization-selected fish (see Supplementary Tables S9-S10). Source data are provided as a Source Data file.

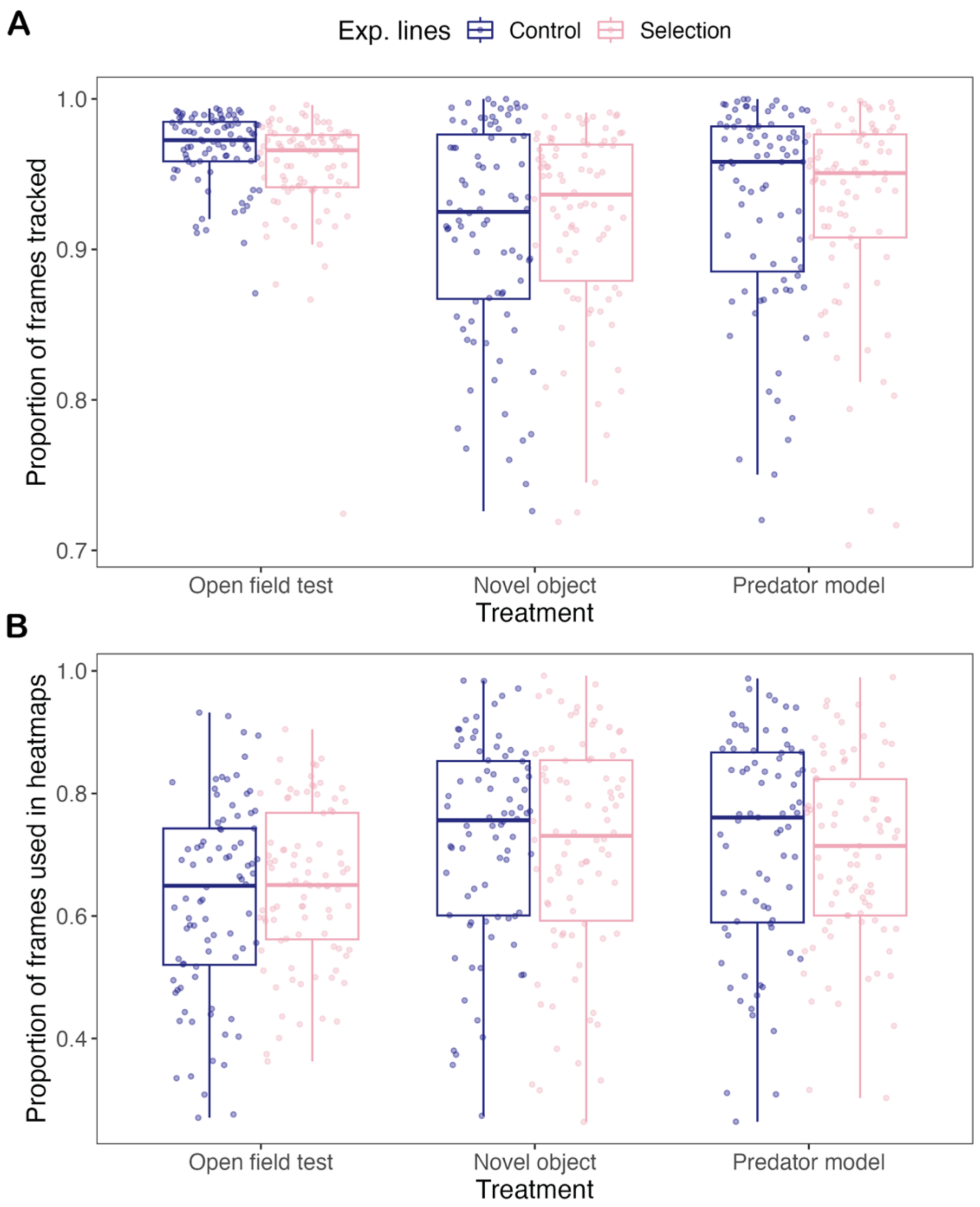

Supplementary Figure 3. Tracking data reliability between groups. We found no significant differences from independent contrasts across treatments obtained from Linear Mixed Models evaluating differences between polarization-selected groups ($n = 89$, pink) and control groups ($n = 85$, blue) in **(A)** the total proportion of frames tracked by idTracker (Estimate $_{\text{Open field}}$: -0.007 ± 0.009, $t = -0.83$, $p = 0.40$; Estimate $_{\text{Novel object}}$: 0.012 ± 0.008, $t = 1.42$, $p = 0.16$; Estimate $_{\text{Predator}}$: 0.001 ± 0.008, $t = 0.15$, $p = 0.88$), or in **(B)** the data used to generate heatmaps that only considered frames in which at least six individuals formed a connected group, with an interindividual distance of 10cm counting as a connection (Estimate $_{\text{Open field}}$: -0.024 ± 0.024, $t = -0.98$, $p = 0.32$; Estimate $_{\text{Novel object}}$: 0.013 ± 0.024, $t = 0.54$, $p = 0.59$; Estimate $_{\text{Predator}}$: 0.012 ± 0.025, $t = 0.49$, $p = 0.62$). For all boxplots, horizontal lines indicate medians, boxes indicate the interquartile range, and whiskers indicate all points within 1.5 times the interquartile range. Source data are provided as a Source Data file.
