# Supplementary material for: Evolution of schooling drives changes in neuroanatomy and motion characteristics across predation contexts in guppies
Source: Nat Commun. 2023 Sep 27;14:6027. doi: 10.1038/s41467-023-41635-6 (PMC10533906; doi:10.1038/s41467-023-41635-6)
Supplement: Supplementary file 3 — Reporting Summary [file 41467_2023_41635_MOESM3_ESM.pdf]

nature portfolio

Corresponding author(s): Alberto Corral Lopez

Last updated by author(s): Sep 5, 2023

Reporting Summary

Nature Portfolio wishes to improve the reproducibility of the work that we publish. This form provides structure for consistency and transparency in reporting. For further information on Nature Portfolio policies, see our Editorial Policies and the Editorial Policy Checklist.

Statistics

For all statistical analyses, confirm that the following items are present in the figure legend, table legend, main text, or Methods section.

n/a	Confirmed	
☐	☒	The exact sample size (*n*) for each experimental group/condition, given as a discrete number and unit of measurement
☐	☒	A statement on whether measurements were taken from distinct samples or whether the same sample was measured repeatedly
☐	☒	The statistical test(s) used AND whether they are one- or two-sided<br>*Only common tests should be described solely by name; describe more complex techniques in the Methods section.*
☐	☒	A description of all covariates tested
☐	☒	A description of any assumptions or corrections, such as tests of normality and adjustment for multiple comparisons
☐	☒	A full description of the statistical parameters including central tendency (e.g. means) or other basic estimates (e.g. regression coefficient) AND variation (e.g. standard deviation) or associated estimates of uncertainty (e.g. confidence intervals)
☐	☒	For null hypothesis testing, the test statistic (e.g. *F*, *t*, *r*) with confidence intervals, effect sizes, degrees of freedom and *P* value noted<br>*Give P values as exact values whenever suitable.*
☐	☒	For Bayesian analysis, information on the choice of priors and Markov chain Monte Carlo settings
☐	☒	For hierarchical and complex designs, identification of the appropriate level for tests and full reporting of outcomes
☐	☒	Estimates of effect sizes (e.g. Cohen's *d*, Pearson's *r*), indicating how they were calculated

Our web collection on statistics for biologists contains articles on many of the points above.

Software and code

Policy information about availability of computer code

Data collection	Collection motion : idTracker (v2.1)<br>Inspection behavior & visual capacities: BORIS behavioral software (v.7.12.2)<br>Eye morphology: ImageJ (v1.53b)<br>Neuroanatomy: NRecon (Bruker microCT)
Data analysis	R (v4.1.3), RStudio (v2022.07.0), Matlab Compiler Runtime 8.3, Matlab (v2020a)<br>Code needed to evaluate the conclusions in the paper are deposited in figshare database under accession code 10.6084/m9.figshare.24080994

For manuscripts utilizing custom algorithms or software that are central to the research but not yet described in published literature, software must be made available to editors and reviewers. We strongly encourage code deposition in a community repository (e.g. GitHub). See the Nature Portfolio guidelines for submitting code & software for further information.

Data

Policy information about availability of data

All manuscripts must include a data availability statement. This statement should provide the following information, where applicable:

- Accession codes, unique identifiers, or web links for publicly available datasets
- A description of any restrictions on data availability
- For clinical datasets or third party data, please ensure that the statement adheres to our policy

Data needed to evaluate the conclusions in the paper are deposited in figshare database under accession code 10.6084/m9.figshare.24080994. Source data are provided with this paper. Additional data related to this paper (video recordings) will be provided upon request from the authors.

Research involving human participants, their data, or biological material

Policy information about studies with human participants or human data. See also policy information about sex, gender (identity/presentation), and sexual orientation and race, ethnicity and racism.

Reporting on sex and gender	N/A
Reporting on race, ethnicity, or other socially relevant groupings	N/A
Population characteristics	N/A
Recruitment	N/A
Ethics oversight	N/A

Note that full information on the approval of the study protocol must also be provided in the manuscript.

Field-specific reporting

Please select the one below that is the best fit for your research. If you are not sure, read the appropriate sections before making your selection.

☐ Life sciences ☐ Behavioural & social sciences ☒ Ecological, evolutionary & environmental sciences

For a reference copy of the document with all sections, see nature.com/documents/nr-reporting-summary-flat.pdf

Ecological, evolutionary & environmental sciences study design

All studies must disclose on these points even when the disclosure is negative.

Study description

We performed a series of experiments in three replicated lines of female guppies artificially selected for higher coordinated motion. For each replicated selection line, we included a control treatment with female guppies that underwent similar environment and experiments, but were not selected based their coordinated motion characteristics.

Research sample

Collective motion: 174 groups of eight female guppies. Fish groups were formed by individuals from the same replicated line, kept in same-sex groups since early signs of maturation and tested when they were between 6-9 months old. We tested these fish in an open field test, exposed to a novel object and to a predator model. Number of trials were balanced per line (over 25 trials per line).
Predator inspection: We used video recordings from collective motion analyses in which groups were exposed to a predator model to score predator inspection within these groups
Visual capacities: We designed an experiment to measure eye morphology, optomotor response and temporal resolution tracking movement to 120 female guppies (20 individuals per line). Fish were kept in same-sex groups since early signs of maturation and tested between 9-12 months old.
Neuroanatomy: We assessed neuroanatomical features of 15 polarization-selected and 15 control fully-grown females (6 months old), divided equally across polarization-selected and control lines.

Sampling strategy

Collective motion & Predator inspection: Sample size (174 groups of 8 fish) was determined by lab space availability and the possibility to test fish groups at similar age (max 3-month difference). Evaluation of collective motion patterns with similar number of tests in these fish (Kotrschal, Szorkovsky et al., Sci Adv 2020) showed strong differences in polarization between selected and control lines.
Visual capacities: We compared 60 polarization-selected versus 60 control female guppies (20 per line). A previously established protocol in the lab (Corral-Lopez et al., 2017 Beh Ecol Sociob) allowed us to establish strong differences in optomotor response and eye morphology using the same numbers when comparing small-brained and large-brained individuals of this species.
Neuroanatomy: We compared 15 polarization-selected versus 15 control female guppies (5 per line). We followed a previously established protocol in the lab to compare neuroanatomical features of large-brained and small-brained guppies (Kotrschal et al. 2017, Evolution) where similar number of fish were used. MicroCT scanning is an invasive technique in this species and we aimed to

find a balance between accurately accounting for individual variation across treatments and ethical principles of reducing the number of samples.

Data collection	Collective motion & predator inspections: Groups of eight sexually mature female guppies were evaluated in an experimental arena in an open field test. After completion of the field test, a novel object or a predator model was incorporated in the setup. The order of presentation of these two treatments was randomized across control and polarization-selected groups. An even number of up to 16 groups of fish were tested per day in our experimental setup, balancing the number of polarization-selected and control groups per day. Composition of groups was uniform in relation to treatment line. All trials were recorded and we tracked positional data of fish from video recordings. Collective motion patterns were extracted from tracking data. Predator inspection behavior was obtained scoring behavior in video recordings during predator model trials. Experiments were performed by A.K and A. F-E. Video analysis was performed by A. C-L.<br>Visual capacities: 120 sexually mature female guppies of same age (20 per line) were randomly selected from the lab population and isolated. Individuals were identified by running numbers. We video recorded their performance in tests designed to measure optomotor response evaluating visual acuity and temporal resolution. Recordings for visual acuity experiments were scored manually using a behavioral scoring software. We tracked positional data of fish from video recordings to obtain temporal resolution tracking movement. Order of video recording was randomized based on identification number. When visual acuity and temporal resolution trials were finished, we photographed fish to obtain eye morphological measurements. Experiments, measurements from images and and video analyses were performed by M.G-O.<br>Neuronatomy: Following fish euthanizing, brain samples were prepared for scanning. Samples for all treatments were prepared in parallel. Running numbers were assigned to each sample. Samples were prepared by A.K.
Timing and spatial scale	Collective motion and predator inspection: To measure groups of fish at similar age, we run experiments in three different collection periods corresponding to each polarization-selected and control replicate pair (Rep1: Jan-Feb 2018; .Rep1: March-April 2018; .Rep1: May-June 2018) .<br>Visual performance: measurements and experiments were performed in a random order during a single collection period (Autumn 2019).<br><br>All experiments were performed in lab facilities at Department of Zoology, Stockholm University (Sweden).
Data exclusions	Collective motion and predator inspection: Following automated tracking, all trials that did not present more than 70% completeness of tracks were disregarded for further analyses. Calculations of median global alignment in each frame of tracking data were only calculated if six out of the eight members of the group presented tracks. To evenly compare motion patterns when presented with a novel object and a predator model to those obtained during the open field assays, we limited our analysis of the open field assay data to the initial six minutes of the recording. For polarization heatmaps and positional data in relation to predator model / novel object, we limited the analyses to frames in which at least six individuals formed a connected group, with an interindividual distance of 10cm counting as a connection. Grid cells that did not contain values for a minimum of 8 groups per treatment were disregarded.<br><br>Predator inspections: Trials that did not present more than 70% completeness of tracks in novel object or predator model treatments were disregarded from behavioral quantification (n = 10).<br><br>Visual performance: From our starting sample size (60 polarization-selected and 60 control individuals), we performed the following number of trials due to handling errors and mortality during the course of the experiments: a) visual acuity test: 59 polarization-selected and 57 control individuals; b) temporal resolution test: 58 polarization-selected and 55 control individuals; c) eye morphology measurements: 57 polarization-selected and 55 control individuals.<br><br>Neuroanatomy: Two brains from polarization-selected lines were damaged during the preparation of samples phase and were excluded for further data collection.
Reproducibility	All experiments performed to polarization-selected and control lines relied on protocols successfully applied in previous investigations in this model species. There were no attempts to repeat the experiments. Trials with incomplete data were excluded as stated above.
Randomization	Fish used in F0 of our artificial selection procedure were a mix of adult fish of various ages from the lab breeding stock. In subsequent generations we used relatively uniform young adults to minimize time between generations. This design ensured that within every replicate, polarization and control fish were of same age. Fish used for experiments in this study are offspring of similar age from selection and control lines of F3 generation.
Blinding	Collective motion, temporal resolution and neuroanatomy data: Data acquisition was based on extraction from positional data or brain region detection using an automated protocol.<br>Predator analyses, visual acuity and eye morphology: Behavioral scoring from videos and measurements from images were blinded by assigning running numbers to files and randomizing the order.

Did the study involve field work? ☐ Yes ☒ No

Reporting for specific materials, systems and methods

We require information from authors about some types of materials, experimental systems and methods used in many studies. Here, indicate whether each material, system or method listed is relevant to your study. If you are not sure if a list item applies to your research, read the appropriate section before selecting a response.

Materials & experimental systems

n/a	Involved in the study
☒	☐ Antibodies
☒	☐ Eukaryotic cell lines
☒	☐ Palaeontology and archaeology
☐	☒ Animals and other organisms
☒	☐ Clinical data
☒	☐ Dual use research of concern
☒	☐ Plants

Methods

n/a	Involved in the study
☒	☐ ChIP-seq
☒	☐ Flow cytometry
☒	☐ MRI-based neuroimaging

Animals and other research organisms

Policy information about studies involving animals; ARRIVE guidelines recommended for reporting animal research, and Sex and Gender in Research

Laboratory animals	Guppies used in our experiment are laboratory-raised descendants of Trinidad guppies sampled from the high predation populations of the Quare River (Trinidad). Generation 0 of selection was a mix of adult fishes of various ages from the laboratory breeding stock, while for the next generations we used relatively uniform young adults (approximately 4-5 months-old). We subsequently used young adults (4-6 months-old) in all experiments and measurements performed in this study.
Wild animals	The study did not involve wild animals
Reporting on sex	We used female guppies as the target of directional selection for higher coordination. Subsequent studies presented here focus only on female guppies.
Field-collected samples	The study did not involve samples collected from the field
Ethics oversight	All experiments were performed in accordance with ethical applications approved by the Stockholm Ethical Board (Dnr:C50/12, N173/13, and 223/15). These applications are consistent with the Institutional Animal Care and Use Committee guidelines.

Note that full information on the approval of the study protocol must also be provided in the manuscript.
